# Detection of multidrug-resistant Shiga toxin-producing *Escherichia coli* in some food products and cattle faeces in Al-Sharkia, Egypt: one health menace

**DOI:** 10.1186/s12866-023-02873-2

**Published:** 2023-05-12

**Authors:** Hanady G. Nada, Amera Saeed El-Tahan, Gamal El-Didamony, Ahmed Askora

**Affiliations:** 1 Drug Radiation Research Department, National Center for Radiation Research and Technology, Egyptian Atomic Energy Authority (EAEA), Cairo, 11787 Egypt; 2 Microbiology and Chemistry Department, Faculty of Science, Zagazig University, Zagazig, 44519 Egypt; 3 Botany and Microbiology Department, Faculty of Science, Zagazig University, Zagazig, 44519 Egypt

**Keywords:** Zoonotic Pathogen, Most probable number technique, Multiple antibiotic resistance index, Virulence factor, STEC non-O157, Biorisk

## Abstract

**Background:**

Shiga toxin-producing *Escherichia coli* (STEC) is a zoonotic pathogen, that is transmitted from a variety of animals, especially cattle to humans via contaminated food, water, feaces or contact with infected environment or animals. The ability of STEC strains to cause gastrointestinal complications in human is due to the production of Shiga toxins (sxt). However, the transmission of multidrug-resistance STEC strains are linked with a severity of disease outcomes and horizontal spread of resistance genes in other pathogens. The result of this has emerged as a significant threat to public health, animal health, food safety, and the environment. Therefore, the purpose of this study is to investigate the antibiogram profile of enteric *E. coli* O157 isolated from food products and cattle faeces samples in Zagazig City, Al-Sharkia, Egypt, and to reveal the presence of Shiga toxin genes *stx*1 and *stx*2 as virulence factors in multidrug-resistant isolates. In addition to this, the partial 16S rRNA sequencing was used for the identification and genetic recoding of the obtained STEC isolates.

**Results:**

There was a total of sixty-five samples collected from different geographical regions at Zagazig City, Al-Sharkia—Egypt, which were divided into: 15 chicken meat (C), 10 luncheon (L), 10 hamburgers (H), and 30 cattle faeces (CF). From the sixty-five samples, only 10 samples (one from H, and 9 from CF) were identified as suspicious *E. coli* O157 with colourless colonies on sorbitol MacConkey agar media with Cefixime- Telurite supplement at the last step of most probable number (MPN) technique. Eight isolates (all from CF) were identified as multidrug-resistant (MDR) as they showed resistance to three antibiotics with multiple antibiotic resistance (MAR) index ≥ 0.23, which were assessed by standard Kirby-Bauer disc diffusion method. These eight isolates demonstrated complete resistance (100%) against amoxicillin/clavulanic acid, and high frequencies of resistance (90%, 70%, 60%,60%, and 40%) against cefoxitin, polymixin, erythromycin, ceftazidime, and piperacillin, respectively. Those eight MDR *E. coli* O157 underwent serological assay to confirm their serotype. Only two isolates (CF8, and CF13), both from CF, were showed strong agglutination with antisera O157 and H7, as well as resistance against 8 out of 13 of the used antibiotics with the highest MAR index (0.62). The presence of virulence genes Shiga toxins (*stx1* and *stx2*) was assessed by PCR technique. CF8 was confirmed for carrying *stx2*, while CF13 was carrying both genes *stx1,* and *stx2.* Both isolates were identified by partial molecular 16S rRNA sequencing and have an accession number (Acc. No.) of LC666912, and LC666913 on gene bank. Phylogenetic analysis showed that CF8, and CF13 were highly homologous (98%) to *E. coli* H7 strain, and (100%) to *E. coli* DH7, respectively.

**Conclusion:**

The results of this study provides evidence for the occurrence of *E. coli* O157:H7 that carries Shiga toxins *stx1* and/or *stx2*, with a high frequency of resistance to antibiotics commonly used in human and veterinary medicine, in Zagazig City, Al-Sharkia, Egypt. This has a high extent of public health risk posed by animal reservoirs and food products with respect to easy transmission causing outbreaks and transfer resistance genes to other pathogens in animal, human, and plants. Therefore, environmental, animal husbandry, and food product surveillance, as well as, clinical infection control, must be strengthened to avoid the extra spread of MDR pathogens, especially MDR STEC strains.

## Background

Shiga toxin-producing *Escherichia coli* (STEC) is considered as an important foodborne zoonotic pathogen worldwide that causes gastrointestinal complications in humans [1]. Healthy domestic ruminants such as cattle, sheep, goats, and pigs can harbor STEC and *E. coli* O157:H7 in their faeces and are thus, natural reservoirs of these pathogens. Other animals, such as, chicken, dogs, horses, etc. are considered as spillover hosts that are susceptible to STEC colonization and can transmit disease [2]. Humans are sporadically infected with STEC that emerged as foodborne pathogens through the ingestion of food, water, or vegetables contaminated with animal faeces, or by direct contact with infected animals [3]. Then, STEC infections are easily transmitted from person-to-person [4]. Diseases caused by STEC isolates, especially serotype O157:H7, ranges from mild diarrhoea to haemorrhagic colitis (HC) and haemolytic uraemic syndrome (HUS). These complications typically affect children, elderly, and immunocompromised patients [5]. The systemic illness of HC, which naturally follows gastrointestinal infections with STEC, typically begins with abdominal cramps and diarrhea, followed by bloody diarrhea. Additionally, HUS which is characterized by acute renal failure, noon-bloody diarrhea converted to bloody diarrhea after one to five days in almost 80% of both, children and adult cases [6]. Due to the severity and pathogenicity of STEC-associated diseases, hospitalization is frequently required and may lead to morbidity and mortality in more than 2–10% of cases [7, 8]. This is mainly attributed to Shiga toxin (Stx) production, as a main virulence factor in STEC [5]. Shiga toxins (Stx) have two major families Stx1 and Stx2, with 70 percent similarity at the amino acid level [9]. Both toxins produce diseases in people, Stx1 is produces less serious diseases linked to human illness, while Stx2 is more often linked with the development of HC and HUS [10]. STEC refers to *E.coli* pathotype capable of producing Stx1, and/or Stx2 [11]. The Shiga toxins mode of actions starts when the pentamer of matching B subunits from Stx permits the toxin to bind to globotriaosylceramide (Gb3) on the host cell [12]. Then, Shiga toxins A subunit inhibits protein synthesis by removing an adenosine residue from the 28S rRNA of the host cell 60S ribosome. Both epithelial and endothelial cells intoxicated with Stx may undergo an apoptotic cell death after intoxication [13, 14]. Moreover, Stx acts on the host cell signal transduction and immune modulation, causing infections [15].

Remarkably, different serotypes of *E. coli* strains are capable of producing Shiga toxins and are associated with diseases worldwide [16]. O157 and non‐O157 STEC strains have been isolated from cases of sporadic diarrhoea and HUS with varying frequencies [11]. Johnson et al. 1996 [17] reported that a quarter of HUS cases in USA were caused by non-O157 STEC. Both, O157 and non-O157 STEC can cause outbreaks attributed to the consumption of contaminated food products, in particular, those derived from cattle, or consumption of water, or vegetables contaminated with ruminant faeces, or direct contact with infected animals [18].

Antimicrobial resistance is a global public and animal health problem, with negative impacts on environmental, individual, social and economic levels [19]. The term multidrug-resistant (MDR) refers to strains that have shown resistance to more than two antibiotics from different antibiotic groups. This is an unfortunate phenomenon that leads to depletion of treatments [20]. Although antibiotic therapy is not a treatment option for human infections caused by STEC O157:H7, MDR strains of STEC O157:H7 or other serotypes (non-O157) should be of great concern. This is because they can easily transfer resistance genes horizontally to other pathogens in hosts and environment [21]. Retail animal food products act as an important vehicle for community wide dissemination of MDR STEC strains. Egypt is part of the Middle East, this geographical region has the maximum annual incidence rates of human STEC cases (122:249 cases/ 100,000 people/ year; 160 HUS cases) compared to other regions worldwide [22]. Thus, if MDR STEC strains are not detected early and addressed, this will definitively lead to serious health problem concerns due to the levitation in the cost of managing infections, and limitation of antibiotic options and emergence of superbugs (MDR strains) in the environment including the aquatic ecosystem. To the best of our knowledge, there is no existing data about the presence of MDR isolates of STEC O157:H7 and non-O157 isolated from both, retail animal food products and cattle faeces in Al-sharkia-Egypt. Therefore, the purpose of the present study was to determine the approximate percentage of *E. coli* O157:H7 and the other serotypes present in the selected food products and cattle’s faeces collected from different geographical regions at Zagazig City, Al-Sharkia-Egypt. Additionally, their susceptibility to widely used antibiotics in human and animal husbandry was evaluated, and it was determined if the highly resistant isolates carry the virulence factors of Shiga toxins genes. Finally, molecular identification for the highly MDR *E. coli* O157:H7 that carries Shiga toxin genes based on partial sequence of 16S rRNA, record the accession number with determination of phylogenetic tree was recorded.

## Results

**Fig. 1:**
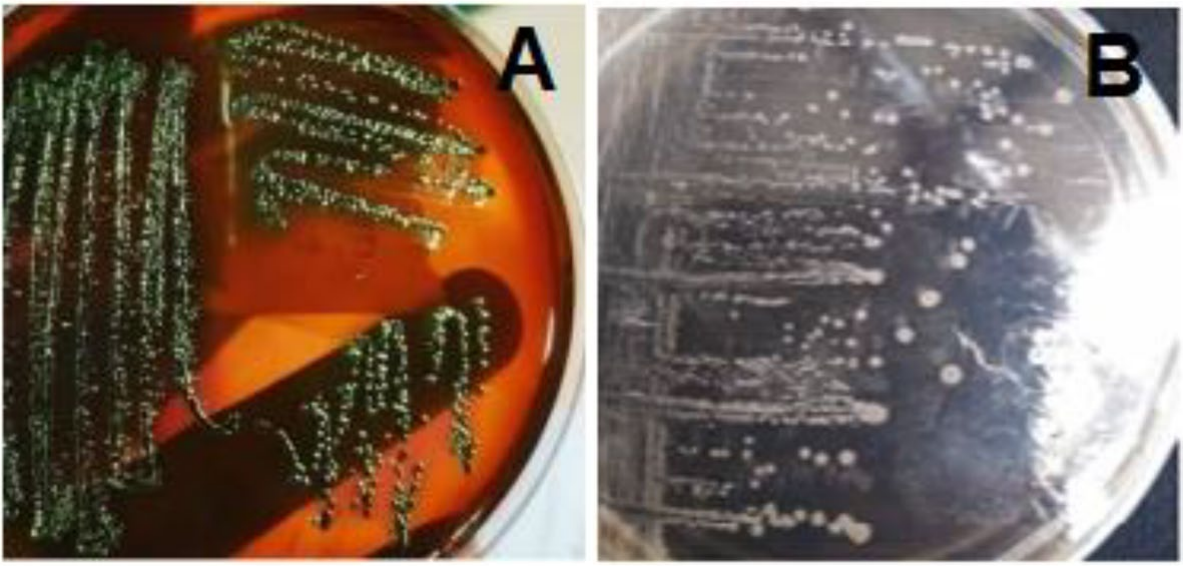
Colonies of *E. coli *on different used selective differential media. **A** on eosin methylene blue (EMB) agar media with metallic sheen appearance, and **B** on sorbitol MacConkey agar media with Cefixime-Telurite supplement (CT-SMAC) with pale (colourless) appearance

The most probable number (MPN) method is a highly used qualitative and quantitative technique for enumerating the fecal coliform bacteria (FCB) in different food, water, animal or human samples [23, 24]. The results of MPN values are illustrated in Table 1. The number of observed positive tubes with gas production or change in media colour indicated the FCB presence in these samples and called presumptive test, Table 2. Out of 65 total samples, in the presumptive test, FCB were isolated from 46%, 60%, 50%, and 100% of chicken meat, luncheon, hamburger and faeces samples, respectively. The prevalence of enteric *E. coli* in the same samples were decreased to 26.7%, 30%, 20%, and 50% after streaking on EMB agar (confirmatory test), as shown in Table 2. After that, only 10 isolates (one isolate from hamburger and 9 isolates from cattle’s faeces) were detected and identified as *E. coli* of serotype O157 by using selective differential sorbitol MacConkey agar media with Cefixime- Telurite supplement (CT-SMAC) in a complete test. Figure 1 (A & B) showed metallic sheen appearance and colourless colonies on EMB, and CT-SMAC, respectively.

**Table 1:** Most probable number (MPN) values per 10 ml of sample and 95% confidence limits for various combination for the 35 food samples at 0.1, 0.01 and 0.001 g/ml dilutions

Samples	No.of tubes giving positive reaction at dilutions (g/ml)			MPN Index per (ml)	Confidence 95% limits	
	**0.1**	**0.01**	**0.001**		**Lower**	**High**
C1	3	2	1	150	37	420
C2	1	0	0	3.6	0.17	18
C3	2	1	0	15	3.7	42
C4	2	3	1	36	8.7	94
C5	1	0	0	3.6	0.17	18
C6	3	3	2	1100	180	4100
C7	3	3	3	> 1100	420	---
C8	3	2	1	150	37	420
C9	3	1	0	43	9.0	180
C10	3	3	0	240	42	1000
C11	1	0	0	3.6	0.17	18
C12	1	1	0	7.4	1.3	20
C13	3	0	0	23	4.6	94
C14	2	2	1	28	8.7	94
C15	1	1	0	7.4	1.3	20
L1	3	3	3	> 1100	420	---
L2	0	0	0	< 0.3	---	0.95
L3	3	1	1	75	17	200
L4	2	3	1	36	8.7	94
L5	3-	2	0	93	18	420
L6	2	2	0	21	4.5	42
L7	3	3	3	> 1100	420	---
L8	3	1	1	75	17	200
L9	3	3	1	460	90	2000
L10	2	0	0	9.2	1.4	38
H1	3	3	1	460	90	2000
H2	2	2	1	28	8.7	94
H3	3	2	1	150	37	420
H4	3	3	3	> 1100	420	---
H5	3	2	2	210	40	430
H6	2	2	2	35	8.7	94
H7	2	1	2	27	+ 8.7	94
H8	3	3	3	> 1100	420	---
H9	3	1	1	75	17	200
H10	2	0	0	9.2	1.4	38

*C* chicken meet, *L* luncheon, *H* hamburger

**Table 2:** Appearance of faecal coliform in positive tubes and identification by using different media

Type of samples	No. of positive samples/ No. of total samples (%)		
	**Presumptive test**	**Confirmatory test**	**Complete test**
**Chicken meat (C)**	7/15 (46.7%)	4/15 (26.7%)	0/15 (0%)
**Luncheon (L)**	6/10 (60%)	3/10 (30%)	0/10 (0%)
**Hamburger (H)**	5/10 (50%)	2/10 (20%)	1/10 (10%)
**Cattle Faeces (CF)**	30/30 (100%)	15/30 (50%)	9/30 (30%)

Presumptive test: number of positive tubes giving acid and gas (change to yellow colour) after incubation, Confirmatory test: number of positive plates giving metallic sheen colony after streaking on EMB media, Complete test: number of positive plates giving colourless white colonies on CT.SMAC

### Antibiotic susceptibility pattern for isolated *E. coli* O157

**Fig. 2:**
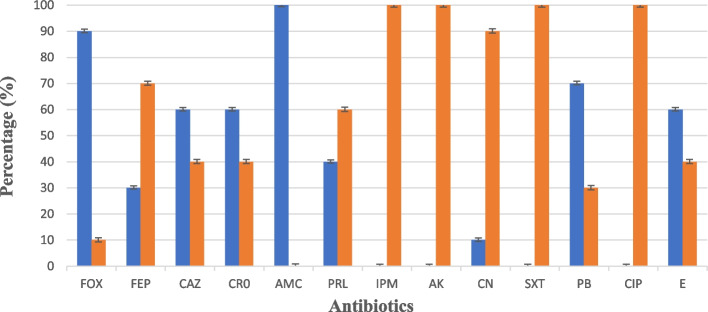
Percentage of resistance and sensitivity of the ten *E. coli *O157 isolates against the 13 chosen antibiotics, FOX: cefoxitin, FEP: cefepime, CAZ: ceftazidime, CRO: ceftriaxon, AMC: amoxicillin/clavulanic acid, PRL: piperacillin, IPM: imipenem, AK: amikacin, CN: gentamicin, SXT: trimethoprim/sulfamethoxazole, PB: polymixin, CIP: ciprofloxacin, and E: erythromycin.

Ten suspected *E. coli* O157 isolates (1 from hamburger sample, and 9 from cattle’s feces samples) were selected and differentiated on CT-SMAC. Then, the isolates were screened for antibiotic susceptibility against the 13 chosen antibiotics, each with different modes of action, and widely used in human and veterinary medicine, Fig. 2 and Table 3. All isolates showed complete resistance (100%) against AMC, with high frequencies of resistance (90%, 70%, 60%,60%, and 40%) against FOX, PB, CAZ, E and PRL, respectively. On the other side, the same isolates showed complete sensitivity (100%) against IPM, AK, and CIP. This means that the higher potency of these antibiotics are able to overcome the pathogenicity of these isolates.

**Table 3:** The phenotypic antibiotic resistance profiles for the *E. coli* O157 isolates

**Code No**	**Diameter of inhibition zone(mm)of different antibiotics**													MAR index
	**β-lactam**							**Aminoglycosides**		**sulfamide**	**polypeptide**	**quinolone**	**Macrolides**	
	**FOX** **(30 μg)**	**FEP** **(30 μg)**	**CAZ** **(30 μg)**	**CR0** **(30 μg)**	**AMC** **(30 μg)**	**PRL** **(10 μg)**	**IPM** **(10 μg)**	**AK** **(30 μg)**	**CN** **(10 μg)**	**SXT** **(25 μg)**	**PB** **(30 μg)**	**CIP** **(5 μg)**	**E** **(15 μg)**	
**CF1**	**R**	S	**R**	**S**	**R**	**I**	**S**	**S**	**S**	**S**	**R**	**S**	**R**	**0.38**
**CF4**	**R**	**I**	**R**	**S**	**R**	**R**	**S**	**S**	**S**	S	**I**	**S**	**I**	**0.31**
**CF5**	**S**	**R**	**R**	**I**	**R**	**R**	**S**	**S**	**S**	**S**	**R**	**S**	**R**	**0.46**
**CF6**	**R**	**S**	**R**	**I**	**R**	**S**	**S**	**S**	**S**	**S**	**S**	**S**	**S**	**0.38**
**CF8**	**R**	**R**	**R**	**R**	**R**	**R**	**S**	**S**	**S**	**S**	**R**	**S**	**R**	**0.62**
**CF9**	**R**	**S**	**I**	**S**	**R**	**S**	**S**	**S**	**R**	**S**	**R**	**S**	**R**	**0.38**
**CF11**	**R**	**S**	**S**	**S**	**R**	**S**	**S**	**S**	**S**	**S**	**R**	**S**	**I**	**0.23**
**CF13**	**R**	**R**	**R**	**R**	**R**	**R**	**S**	**S**	**I**	**S**	**R**	**S**	**R**	**0.62**
**CF15**	**R**	**S**	**I**	**S**	**R**	**S**	**S**	**S**	**S**	**S**	**I**	**S**	**I**	**0.15**
**H5**	**R**	**S**	**I**	**S**	**R**	**S**	**S**	**S**	**S**	**S**	**I**	**S**	**I**	**0.15**
**Antibiotic resistance (R)%**	**90%**	**30%**	**60%**	**20%**	**100%**	**40%**	**Nil**	**Nil**	**10%**	**0%**	**70%**	**Nil**	**60%**	

*CF* cattle faeces, *H *hamburger, *MAR index *multiple antibiotics resistance = No. of antibiotics to which the isolate is resistant / Total No. of used antibiotics, No.: number, R: resistant, S: sensitive, I: intermrdiate, *FOX *cefoxitin, *FEP* cefepime, *CAZ* ceftazidime, *CRO *ceftriaxon, *AMC* amoxicillin/clavulanic acid, *PRL* piperacillin, *IPM* imipenem, *AK* amikacin, *CN* gentamicin, *SXT* trimethoprim/sulfamethoxazole, *PB* polymixin, *CIP* ciprofloxacin, *E* erythromycin

Most of *E. coli* O157 isolates were resistant to the tested antibiotics. Multidruge resistance (defined as resistance to three antibiotic classes or more) was observed in 8 out of 9 faecal samples (89%) with the highest MAR index values (ranged between 0.23 to 0.62). Whereas, only one faecal sample (11%) expressed resistance to two antibiotics; FOX, and AMC, both antibiotics from β-lactam group. The same resistance pattern is observed in the only isolate recovered from hamburger (5H).

Out of 8 suspected *E. coli* O157 isolates, only two (CF8 and CF13) had clearly strong agglutinations with both antisera O157 and H7. This means that CF8 and CF13 were confirmed as STEC O157:H7. Noticeably, both isolates CF8 and CF13 were recovered from cattle faeces and showed the highest MAR index (0.62) amongst all isolates. Both isolates were resistant to 8 antibiotics (FOX, FEP, CAZ, CRO, AMC, PRL, PB, and E) belonging to 3 different groups (β-lactam, polypeptide, and macrolides).

PCR analysis was performed to confirm the presence of the main virulence factor in STEC O157:H7; Shiga toxins (*stx1* (encodes variant 1 [Stx1]), and *stx*_2_ (encodes variant 2 [Stx2]).

### Multiplex PCR for *stx1*, and* stx*2 genes

**Fig. 3:**
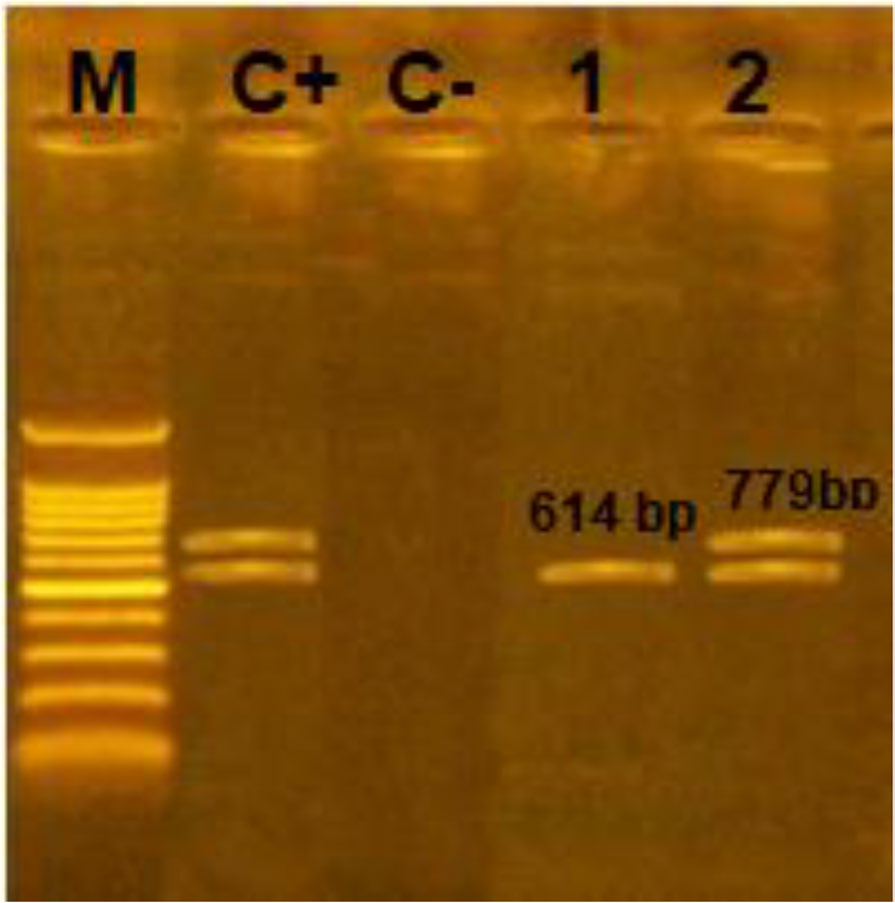
Agarose gel electrophoresis of DNA fragments generated by multiplex PCR for virulence gene- shiga toxins of selected *E. coli *isolates, Lane M: DNA molecular weight marker (100 bp), Lane C+: Positive control, Lane C-: Negative control, Lane1*: E.coli *isolate of code no. CF8, Lane 2: *E.coli *isolate of code no. CF13

PCR analysis was performed to confirm the presence of main virulence factor in both STEC O157:H7 isolates; Shiga toxins genes (*stx1* (encodes variant 1 [Stx1]), and *stx*_2_ (encodes variant 2 [Stx2])). Figure 3 showed that isolate CF8 contains *stx2* gene while isolate CF13 had *stx1* and *stx2* genes.

### Molecular identification of multidrug- resistant bacterial isolates based on partial sequence of 16S rRNA gene

**Fig. 4:**
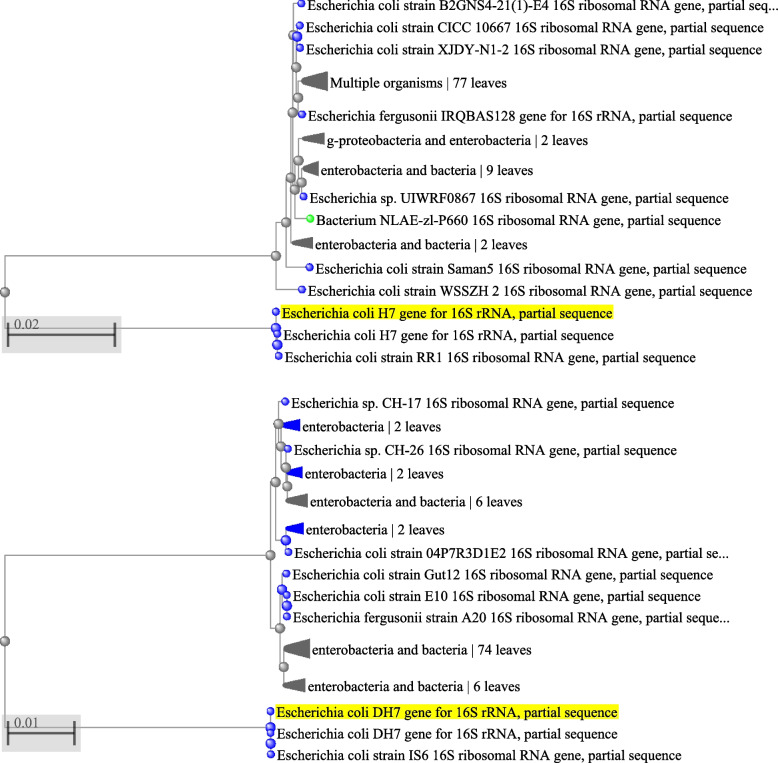
Phylogenetic tree analysis based on 16S rRNA nucleotide sequence alignment for our two *E.coli *O157:H7 isolates with other related members that possess the best similarity. The scale bar at the bottom (left) indicates similarity coefficient (%)

In Table 4, the identification of the two isolates of *E.coli* O157:H7 were confirmed using the 16S rRNA gene. The Gene-Bank nucleotide sequence accession numbers for partial sequences of 16S rRNA gene were generated in this study using DDBJ Center, and recorded as CF8 *Escherichia coli* LC666912 and *Escherichia coli* LC666913. Figure 4 showed the sequence of mono toxin *E.coli* O157:H7 isolate No (CF8) was similar 98% to strain CP024273,CP024269 and CP02422 that were enterotoxigenic *Escherichia coli* Strain *E.coli* O169:H41 [25, 26], and CP041429 which was *Escherichia coli Stx*2 producing strain serotype O100:H19 [27], as well as other strains of *Escherichia coli.* On the other hand, the sequence of di toxin *E.coli* O157:H7 isolate No (CF13) was similar 99% to *Escherichia coli* that cause diarrhea and gastrointestinal disease [28].

**Table 4:** Identification of the two *E.coli* O157:H7 isolates, conformed by 16S rRNA gene with Gene-Bank nucleotide sequence accession numbers

Isolates No	Definition	Accession numbers
CF8	*Escherichia coli H7*	LC666912
CF13	*Escherichia coli* DH7	LC666913

## Discussion

Cattle are considered to be the principal reservoirs of pathogenic *E. coli* O157:H7 causing environmental hazards and human diseases, and transmission may occur through a variety of routes[29]. For example, the bovine faeces can contaminate crops, and drinking or recreational water intended for human consumption, animal contact, and person-to-person spread [30–32]. Additionally, consumption of contaminated raw/ undercooked meat or poultry products as hamburgers and luncheon is considered amongst the major routes for STEC O157:H7 and other serotypes transmission as a zoonotic foodborne pathogen [33, 34]. Our local results in Zagazig City, Al-Sharkia, Egypt indicated that, 10 isolates (1 from hamburger sample, and 9 from cattle’s faeces samples) out of 65 total samples (representing 15.4%) were highly suspected to be *E. coli* O157 by using EMB, and CT-SMAC media. However, *E.coli* ferment lactose and sucrose appear as metallic green sheen colony on EMB [35]. While, SMAC medium specific for isolation of non-fermented sorbitol *E.coli* O157 [36], and supplemented with cefixime and potassium tellurite to increase the selectivity of *E.coli*O157 isolation [37]. SMAC medium have high specificity for detection of *E.coli* O157.

Our results revealed high values of multiple antibiotic resistance (MAR) index for all suspected *E. coli* O157 isolates (ranged between 0.15 and 0.62). These values are higher than the accepted international standard for MAR index [38]. Whereas, the MAR index is calculated as the ratio between the number of antibiotics to which an organism is resistant / the total number of antibiotics to which the organism is exposed. For that, bacteria having MAR index ≥ 0.2 mainly originate from a high-risk source of contamination in which several antibiotics are used. Eighty % is the resistance percentage among our local suspected *E.coli* O157 isolates, that showed resistance against two or more antibiotics having different mode of actions, and can be classified as multidrug-resistant (MDR) isolates. This percentage of resistance among our local suspected *E.coli* O157 isolates were significantly higher than the isolates of the *E. coli* O157:H7 from cattle (53%) and beef (57%) reported by [39] from another geographic region (Spain, Europe). Among all the isolates, antibiotic resistance was 100%, 90%, 70%, 60%, and 60% against AMC, FOX, polymyxin B (PB), CAZ, and erythromycin (E), respectively. These results are consistent with previous study [40]. Several studies have reported complete resistance (100%) against AMC with 70% resistance against FOX [41, 42]. Meanwhile, other studies demonstrated a lower percentage of resistance (45.5%, and 36.4%) against AMC and PB, respectively, with complete resistance (100%) against E, in Malaysian and Korean *E.coli* O157 isolated from food and animal sources [43]. The same results were reported in poultry samples from Saudi Arabia [44] and from Bangladesh [45]. All antibiotics tested here are considered to be critically or highly important for human medicine by the World Health Organization, and commonly used in Egypt [46] Nevertheless, nearly all classes of antibiotics offered to humans have also been used in animal husbandry [47]. The same antibiotics used in this study were reported to be used to treat respiratory infections, diarrhoea, mastitis, and other infections in beef and dairy cattle [48]. In Egypt, several antibiotics have been recorded to be widely used in animal husbandry for several reasons, for example: to guarantee healthy livestock before slaughtering, avoid infections, and promote animal growth. Such indiscriminate use of antibiotics will lead to presence of resistance pathogens in faeces and in animal tissues, which may be easily widespread to other creatures [20]. This may strongly explain the high prevalence of MDR amongst our local suspected *E.coli* O157 collected from food products and cattle faeces. Katsuda et al., 2009 [49] reported that antimicrobial resistance has been increasingly identified in animal pathogens.

It is noteworthy in this study that the most frequent source for MDR *E.coli* O157 in Al-Sharkia, Egypt that may cause environment and public health risk, was the CF samples. Where, eight out of the nine (89%) CF samples that were suspect to have *E.coli* O157 isolates had the highest MAR index (ranged between 0.23- 0.62), and were considered as MDR. Our local results suggest that CF samples are the leading cause of pathogenic *E. coli* O157 contamination. Wang et al. [50] pointed out that the bovine faeces are a potential source for the spread of STEC strains to the human food chain, as well as to the environment. The effective control of STEC strains mandate correct handling and usage of bovine (cattle) faeces to decrease the risk of contamination of the environment and food by this pathogen. Even more so, by the presence of many reports for long-term survival of both *E. coli* O157 and non-O157 in faeces of dairy and beef cattle [50].

Two isolates out of our local suspected *E. coli* O157, were both obtained from CF samples (CF8, and CF13), and confirmed as *E.coli* O157:H7 by serological test, as they gave strong agglutination with O157 antigen and H7 antigen. The agglutination test appeared specific for the diagnosis of *E. coli* O157:H7, these results are in agreement with, [51, 52], who used latex agglutination test for serotyping of *E. coli* O157:H7. It is a rapid, reliable test that is easy to perform.

Our results revealed that, the prevalence of *E.coli* O157:H7 in Al-Sharkia, Egypt (two out of 30 samples) is equal to 6.7%. This ratio is similar to results that were obtained from sheep faeces in another Egypt governorate (Giza) [53], which is close to the prevalence of *E. coli* O157 in sheep faeces at Pastures in Scotland (6.5%) [54], and 7.1% in Northern Spain [55]. Nonetheless, our ratio for prevalence of *E.coli* O157 in Al-sharkia, Egypt is clearly higher than that recorded in South Yorkshire, UK (1–2%) and Spain (2.6%) by [23, 56], respectively, and also higher than in Great Britain, Spain, and Switzerland as recorded [57–59], respectively.

Pathogenicity of *E.coli* O157:H7 is associated with different virulence factors, production of Shiga toxins (stx1 and stx2) being the most potent one [60, 61] . Louise et al. [62] reported that stx1 and stx2 cause different grades and types of tissue damage, and stx2 is more toxic than stx1 to renal endothelial cells in mammals. Among our two tested MDR *E.coli* O157:H7 isolates, CF8 was found to carry *stx*2 gene, and CF13 carry *stx*1 and *stx*2 genes; this is according to molecular analysis by multiplex PCR.

Particularly, as *E. coli* O157 can survive in the environment for more than 10 months, as reported by [63], humans and animals could be at risk of infection long after an environment is initially contaminated. Very low concentrations of STEC O157:H7 can be a source of outbreaks or sporadic infection, as stated by [64]; whom have estimated that the infectious dose for this STEC pathogen to be under 100 colony-forming unit/ patient. Others, estimated the infectious dose to be just 10 bacteria [65]. Occurrence of STEC O157:H7 in local cattle faeces suggest higher direct, or indirect transmission of STEC O157 may lead to outbreaks, due to work of the large population et al.-Sharkia in agriculture and cattle breeding. Direct transmission of STEC O157:H7 via the fecal–oral route, may be from calves to children, manure exposure in the field, or dirty hands. While, indirect transmission could be through: slaughtering, the natural progression of the food chain, or from contaminated soil, water, or vegetables with cattle faeces.

Shiga toxin-producing *E. coli* (STEC) has been deeply involved in foodborne, waterborne and airborne outbreaks in several studies all over the world [66], and have demonstrated that the outbreak of *E. coli* O157:H7, caused the infection for 20 campers out of 337 at scout camp on May 2000, it was due to an environmental source. Investigations do not suggest that it was a food vehicle, as they did not find any *E.coli* isolates in food or drinking water. However, investigations supported that the transmission of *E. coli* O157:H7 was due to exposure to the contaminated environment by hands, either straightly from hand to mouth or via food. They found that the food in studied cases worked as an inert vehicle for *E. coli* O157:H7 transmission, not as a growth medium (source of infection) for the bacterial pathogen. The contamination of the environment may be due to the fact that the camp took place on an agricultural showground used for sheep grazing. Additionally, the location of the camp had been fully contaminated with sheep faeces.

The isolated toxigenic O157 *E.coli* strains carrying *stx*1 and *stx*2 genes mainly showed MDR towards numerous antibiotics that are medically important, as they commonly used in human and animal treatment. There is a high possibility that these resistant strains can endure the environment for a long time and, then, eventually transmit these resistance determinants to other environmental bacteria [67].

Such transfer of resistance determinants could fuel the spread of MDR bacteria, this could have grave implications on the health of humans and animals; thus, increasing the burden of disease in the community. Therefore, there is a great need for urgent policy formulations on the prudent use of antimicrobials in both human and veterinary medicine, because failure in this regard could spell doom in the near future [68].

There are high recommendations from different authorities worldwide for testing all ground meat/poultry samples for STEC O-serotypes, as these organisms are shed in faeces, and will lead to environmental contamination for an extended period of time as the USDA Food Safety and Inspection Service states.

## Conclusions

The importance of recent study is to monitor the prevalence of suspected *E.coli* O157 in Zagazig City et al.-Sharkia, Egypt by 15.4% in grounding beef, as hamburger and cattle faeces samples are of epidemiological significance. Furthermore, there is a high prevalence of MDR (80%) determinants in suspected *E.coli* O157. Accordingly, this will lead to the emergence of superbugs of clinical relevance due to the ease of transfer of resistance genes to other pathogens. In addition, two isolates (6.7%) recovered from cattle faeces were carried the Shiga toxin genes stx1 and/or stx2, which suggests that the farm environment may serve as a channel for the dissemination of antimicrobial-resistant STEC strains to other niches, including the food chain. Risk assessment is needed to protect human, animal, and environmental health. Furthermore, animal faeces should be treated before discharge, continuous monitoring should be considered, and surveillance for environment, livestock, and food product should be strengthened.

## Methods

### Samples collection and preparation

A total of sixty-five samples were collected in the period between Jan. 2021 to March 2021. Out of this, 35 samples (15 chicken meat, 10 luncheon, and 10 hamburger) were collected from diverse markets and butcher’s shops in Zagazig City, AL-Sharkia Governorate- Egypt. While, the remaining 30 samples, were cattle’s faecal samples collected from different house farms, and the Veterinary Medicine in Zagazig University at Zagazig City, AL-Sharkia Governorate- Egypt.

Following the same procedure as [20], all samples were packaged in sterilized polyethylene bags and transferred directly within 1–2 h, in a cold box at 4 °C to the laboratory. Each bag was labeled with the source name and number.

At the laboratory, each sample underwent the following process. Twenty-five grams of the sample was transferred to a sterile flask using sterile spatula, and 225 ml of Tryptone Soya Broth (TSB) was added. Homogenization took place in a clean blender at 2000 r.p.m for 1–2 min. Then, the homogenized sample was transferred into a sterilized flask and incubated at 37 ± 2 °C for 18-24 h.

### Enumeration of faecal coliform bacteria in samples, isolation, and purification of *E. coli* O157

To enumerate the low concentration of the total fecal coliform bacteria FCB (including undetectable *E. coli*) in each sample alone, the three-tubes most probable number (MPN) technique was performed as in the U.S. Food and Drug Administration Bacteriological Analytical Manual, which is well described by [69]. The MPN is a statical assay based on probability theory, and a multi-step assay consisting of three-steps; presumptive test, confirmed test, and completed test [70] based on lactose fermentation. Briefly, at the first screening test (presumptive test), serial dilutions (0.1,0.01,0.001) of each sample were prepared. Then, each dilution was inoculated into three test tubes containing 10 ml of MacConkey broth (MB) (Oxoid, UK), and Durham’s tube. After overnight incubation at 37 ± 2 °C, positive samples the presence of FCB produced gas or changed MB colour from red to yellow. The number of tubes giving a positive reaction (in each dilution) were compared to a standard chart, and the number of FCB present in each sample was determined by using the MPN index (just for the 35 food samples). The Confidence 95% intervals were used to represent the probability of the results that cover the actual concentration before inoculating the tubes, which was at least 95%. Loopful from each positive tube was re-streaked on MacConkey Agar medium (Oxoid, UK) to get pure colonies of FCB isolates.

Then, the confirmatory test was performed by picking up pure colonies of FCB isolates and streaked on eosin methylene blue (EMB) agar medium (oxoid CM69, UK), in order to isolate and differentiate *E. coli* colonies from other FCB isolates. In this test, a loopful from each positive tube was streaked on eosin methylene blue agar medium (EMB) (oxoid CM69, UK). After an incubation period 18-24 h at 37 ± 2 °C, colonies with metallic sheen appearance were picked up and purified as *E. coli* isolates for a completed test.

Purified *E. coli* colonies obtained from previous assays were separately streaked onto selective differential sorbitol MacConkey agar media with cefixime- telurite supplement (CT-SMAC) (Oxoid, UK), which has a selective supplement (cefixime- telurite) for specific *E. coli* O157 isolation. After the incubation period, *E. coli* serogroup O157 colonies had a pale (colourless) appearance due to their inability to ferment the sorbitol [71]. These colonies were picked up, labeled with the source of sample and number, and underwent further investigations in this study.

### Antibiotic susceptibility for *E. coli* O157 isolates

All presumptive purified *E. coli* O157:H7 were tested for their susceptibilities to the selected antibiotics by standard Kirby-Bauer disc diffusion method [72]. Thirteen antibiotic discs (Oxoid, Uk) were chosen as they are commonly used in treatment infections in local hospitals and recommended worldwide [48, 73]. Additionally, they represent different groups of antibiotics with different modes of action against *E. coli* isolates. The 13 chosen antibiotics include the following; cefoxitin (FOX, 30 µg), cefepime (FEP, 30 µg), ceftazidime (CAZ, 30 µg), ceftriaxon (CRO, 30 µg), amoxicillin/clavulanic acid (AMC, 30/10 µg), and imipenem (IPM, 10 µg) belonging to β-lactam antibiotics, piperacillin (PRL, 10 µg), gentamicin (CN, 10 µg), amikacin (AK, 30 µg),trimethoprim-sulfamethoxazole (SXT, 1.25/23.75 µg), ciprofloxacin (CIP, 5 µg), erythromycin (E, 15 µg), and polymixin (Pb30 µg). The test was performed as illustrated by [74] and the results were interpreted as resistant (R), intermediate (I) or sensitive (S), by measuring the diameter of the inhibition zone (DIZ) in mm around each disc in accordance with the recommendation of Clinical and Laboratory Standard Institute (CLSI, 2018). The antibiogram pattern of each isolate against the thirteen tested antibiotics was obtained and the multiple antibiotic resistance (MAR) index was calculated as follows:

1 $$\mathbf M\mathbf A\mathbf R\boldsymbol\;\mathbf i\mathbf n\mathbf d\mathbf e\mathbf x\boldsymbol\;=\;\mathbf N\mathbf o.\;\mathbf o\mathbf f\boldsymbol\;\mathbf a\mathbf n\mathbf t\mathbf i\mathbf b\mathbf i\mathbf o\mathbf t\mathbf i\mathbf c\mathbf s\boldsymbol\;\mathbf{to}\boldsymbol\;\mathbf w\mathbf h\mathbf i\mathbf c\mathbf h\boldsymbol\;\mathbf t\mathbf h\mathbf e\boldsymbol\;\mathbf i\mathbf s\mathbf o\mathbf l\mathbf a\mathbf t\mathbf e\boldsymbol\;\mathbf{is}\boldsymbol\;\mathbf r\mathbf e\mathbf s\mathbf i\mathbf s\mathbf t\mathbf a\mathbf n\mathbf t/\mathbf T\mathbf o\mathbf t\mathbf a\mathbf l\boldsymbol\;\mathbf N\mathbf o.\;\mathbf o\mathbf f\boldsymbol\;\mathbf u\mathbf s\mathbf e\mathbf d\boldsymbol\;\mathbf a\mathbf n\mathbf t\mathbf i\mathbf b\mathbf i\mathbf o\mathbf t\mathbf i\mathbf c\mathbf s$$

Isolates with high MAR index were recorded as multidrug-resistant (MDR) isolates, especially if they showed resistance to more than two antibiotics from different groups [20].

### Serological identification of MDR *E. coli* O157 isolates

According to the antibiogram pattern, the multidrug- resistant (MDR) isolates were serologically identified by slide agglutination according to [75] by using rapid monovalent O157and H7 antisera (DENKA SEIKEN Co., Japan). The isolate showed agglutination in O157 antiserum and H7 antiserum was confirmed as *E. coli* O157:H7, and they were selected to approve their carrying of Shiga toxin genes.

### Amplification of Shiga toxin *stx1* and *stx*2 virulence genes

DNA from *E. coli* O157:H7 isolates gave positive reaction for agglutination test with both anti-O157 and anti-H7 antibodies, and was extracted by using QIAamp DNA Mini kit (Qiagen, Düsseldorf, Germany, GmbH). Next, the isolates were subjected to molecular analysis for the presence of Shiga toxins (*stx1* and *stx*2) as a potential virulence gene in *E. coli* O157:H7, by using multiplex PCR. The primer sequences were designed by [76], as illustrated in Table 5.

**Table 5:** Primer sequences designs for virulence genes of Shiga toxins; *Stx1* and *Stx2* in MDR *E. coli* O157 with the universal 16 s rRNA

Gene	Primer sequence (5'-3')	Length of amplified product	Reference
*stx1*	F- ACACTGGATGATCTCAGTGG R- CTGAATCCCCCTCCATTATG	614 bp	Dipineto et *al., 2006* [76]
*stx2*	F- CCATGACAACGGACAGCAGT T R- CCTGTCAACTGAGCAGCACTTTG	779 bp	
Universal 16 s rRNA	F- AGAGTTTGATCCTGGCTCAG	1038 bp	(Baron et al. 2013) [78]

MDR: multidrug- resistant, *stx*1: encodes Shiga toxin variant [Stx1], *stx*2: encodes Shiga toxin variant 2 [Stx2]

PCR was carried out in a 50 µL reaction containing 25 µL of EmeraldAmp GT PCR mastermix (2 × premix)(Takara, Japan), 1 µL of each primer (20 pmol), 13 µL of water, and 8 µL of DNA template. The reactions were performed in an Applied Biosystems 2720 thermal cycler (Biometra, Göttingen, Germany). The products of PCR were separated by electrophoresis on 1.5% (Wt/Vol) agarose gel (Applichem GmbH, Germany) in 1 × TBE buffer (10.78 g/L Tris buffer, 5.5 g/L Boric acid, 0.82 g/L EDTA, pH 8.3) at room temperature using gradients of 5 V/cm. For gel analysis, 20 µL of the products were loaded in each gel slot. A Gelpilot 100 bp Ladder (Qiagen GmbH, Germany) was used to determine the fragment sizes. The gel was photographed by a gel documentation system (Alpha Innotech, Biometra) and the data was analyzed using associated software [77].

### Polymerase Chain Reaction (PCR) for amplification of 16S rRNA gene

PCR amplification was performed to confirm the identity of *E. coli* O157:H7 using universal 1492r primer [78], as in Table 5.

The extracted bacterial genome must be diluted before PCR performance by adding 495 μl of nuclease free water into 1.5 ml micro centrifuge tube and 5 μl of the supernatant from the PrepMan ultra extracted sample was added to get 1: 100 dilution; then, vortexed and mixed well. Thermo-scientific was used to amplify the first 500 base pairs of the 16S rRNA bacterial gene in the sample. PCR set up was performed by adding the following components: 12.5 μl of Dream Taq Green PCR Master Mix (2X) with Cat. No. K1081, 0.5 μl of Forward Primer (10 pmol), 0.5 μl of Reverse Primer (10 pmol), 10.5 μl Nuclease Free Water and 1.0 μl DNA Template; the tube was closed tightly and placed in the thermal cycler. PCR reaction conditions were initial denaturation at 95 °C for 4 min, 40 cycles at 95 °C for 30 s, 60 °C for 30 s and 72 °C for 40 s. Final extension at 72 °C for 10 min was done. The PCR product was run and visualized by loading 10 μl of the PCR product per lane on 1.8% agarose gel that stained by ethidium bromide against 100 bp DNA ladder as a marker using 1X TAE as a running buffer, and photographed by gel documentation system to identify its size.

### Purification of PCR product

PCR products were purified according to the manufacturing procedure of Gene JET PCR Purification Kit (Thermo Scientific, Cat. No. K0701) as per the following steps: five microliters of the PCR reaction product was mixed with 2 μl of GeneJET reagent for a total of 7 μl of the reaction volume, and incubated at 37 °C for 15 min to degrade the remaining primers and nucleotides. Then, the sample was incubated at 80 °C for 15 min to inhibit the reagent reaction. The purified PCR product stored at -20 °C for sequencing.

### DNA sequencing for the amplified gene

PCR products were sequenced using the standard Sanger method on ABI 3730XL DNA Sequencer at Macrogen sequencing services (Macrogen, Seol, South Korea for forward and reverse sequencing.

### Sequence alignment and phylogenetic analysis

Pair wise and multiple DNA sequence alignment were carried out using CLUSTALW multiple sequence alignment programme version (7.0.9) (http://www.ebi/ac.uk/clustalw) [79]. Bootstrap neighbor joining tree was generated using MEGA version 3.1[80] from CLUSTALW alignment. Comparison with sequences in the Gene Bank database was achieved in BLASTN searches at the National center for Biotechnology Information site (htto:// ncbi. nlm.nih.gov).

The Gene-Bank nucleotide sequence accession numbers for partial sequences of 16S rRNA gene were generated in this study using DDBJ Center [81].

### Statistical analysis

All statistics with a probability value of ≤ 0.05 were considered significant. The bacterial counts were normalized by transforming the data using log10. The MPN estimated by selecting the dilutions of positive tubes, and all the dilutions between them by using [82] formula;

2 $$\mathbf M\mathbf P\mathbf N/\mathbf g=\left(\sum\mathbf g\mathbf j\right)/\left(\sum\mathbf t\mathbf j\mathbf m\mathbf j\sum\left(\mathbf t\mathbf j-\mathbf g\mathbf j\right)\mathbf m\mathbf j\mathbf{({}^{1\!\!}/{}_{2})}\right)$$

where the sum is over the selected dilutions, and ∑gj = the number of positive tubes in the selected dilutions, ∑tjmj = the grams of sample in all tubes in the selected dilutions. The confidence 95% limits for any dilution test calculated by estimating the standard error of log10 (MPN) by the method of [83].

3 $$\mathbf L\mathbf o\mathbf g10\left(\mathbf M\mathbf P\mathbf N\right)\pm1.96\ast\left(\mathbf S\mathbf t\mathbf a\mathbf n\mathbf d\mathbf a\mathbf r\mathbf d\boldsymbol\;\mathbf E\mathbf r\mathbf r\mathbf o\mathbf r\right)$$

The mean value (from the three replicates) was plotted against time by linear regression analysis using cultural detection of *E. coli*. The slope of the line was calculated to obtain a growth rate for the organism (the unit for growth rate is log10 CFU).

## Data Availability

The datasets used and/or analyzed during the current study are available from the corresponding author on request. The sequences of two strains analyzed were deposited in the National Library of Medicine, National Center for Biology Information (NCBI), GenBank nucleotide sequence database. The accession numbers assigned as LC666912 (https://www.ncbi.nlm.nih.gov/nuccore/LC666912) and LC666913 (https://www.ncbi.nlm.nih.gov/nuccore/LC666913).

## Abbreviations

*E. coli*: *Escherichia coli*
STEC: Shiga toxin-producing *Escherichia coli*
stx: Shiga toxins
MDR: Multidrug-resistant
MAR: Multiple antibiotic resistance
MPN: Most probable number
HUS: Hemolytic-uremic syndrome
HC: Hemorrhagic colitis
FCB: Fecal coliform bacteria
C: chicken meat samples
L: luncheon samples
H: Hamburger samples
CF: Cattle faeces samples
CT-SMAC: Sorbitol MacConkey agar media with Cefixime-Telurite supplement
MB: MacConkey broth media
EMB: Eosin methylene blue medium
TSB: Tryptone Soya Broth media
TBE: Tris Borate EDTA buffer
R: Resistant
I: Intermediate
S: Sensitive
DIZ: Diameter of inhibition zone
FOX: Cefoxitin
FEP: Cefepime
CAZ: Ceftazidime
CRO: Ceftriaxon
AMC: Amoxicillin/clavulanic acid
PRL: Piperacillin
IPM: Imipenem
AK: Amikacin
CN: Gentamicin
SXT: Trimethoprim/sulfamethoxazole
PB: Polymixin
CIP: Ciprofloxacin
E: Erythromycin
